# Severe Prenatal Presentation of Adenylosuccinate Lyase Deficiency Caused by a Synonymous *ADSL* Variant Inducing Aberrant Splicing

**DOI:** 10.1002/pd.70087

**Published:** 2026-01-30

**Authors:** Aysegül Klapperich, Vaclava Skopova, Mert Karakaya, Clara Velmans, Christian Netzer, Brigitte Strizek, Lenka Steiner Mrazova, Marie Zikanova

**Affiliations:** ^1^ MVZ Humangenetik Köln Cologne Germany; ^2^ Research Unit for Rare Diseases, Department of Paediatrics and Inherited Metabolic Disorders First Faculty of Medicine Charles University in Prague and General University Hospital in Prague Prague Czech Republic; ^3^ Institute of Human Genetics, Faculty of Medicine and University Hospital Cologne University of Cologne Cologne Germany; ^4^ Institute of Human Genetics Heinrich‐Heine‐University University Hospital Düsseldorf Düsseldorf Germany; ^5^ Department for Obstetrics and Prenatal Medicine University Hospital Bonn Bonn Germany

**Keywords:** aberrant splicing, adenylosuccinate lyase deficiency, ADSL, fetal phenotype, prenatal diagnosis, synonymous variant

## Abstract

- What is already known about this topic?
  ◦ ADSL deficiency is a rare metabolic disorder, typically diagnosed postnatally with variable severity.
  ◦ ADSL activity is known to be reduced in affected patients.
  ◦ Pathogenic variants in *ADSL* are predominantly missense or truncating; no pathogenic synonymous variants have been reported.
- What does this study add?
  ◦ The first functionally confirmed case of severe prenatal‐onset ADSL deficiency is described.
  ◦ The first pathogenic synonymous *ADSL* variant causing aberrant splicing is identified.
  ◦ Reduced ADSL activity in PBMCs of unaffected parents is demonstrated for the first time.

## 1 Fetal Phenotype

**FIGURE 1:**
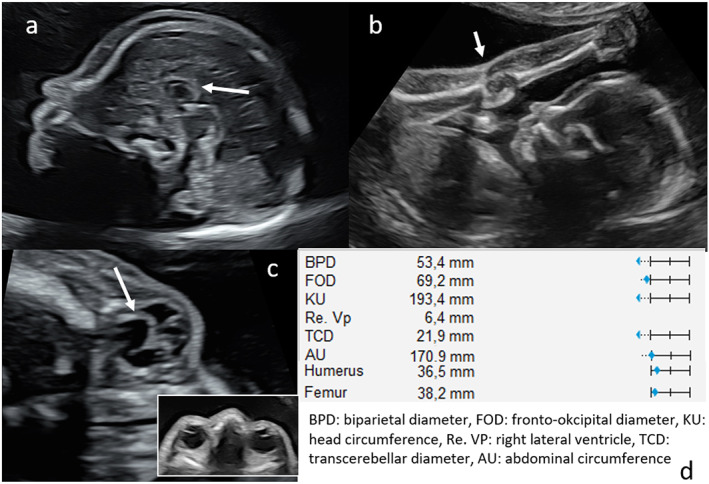
*Sonography at 24 weeks of gestation.* (a) Dysplastic short corpus callosum (arrow), hypoplastic brain stem (b) legs in fixed extension at the knees (arrow) in front of fetal body, (c) intraocular anomaly (retinal detachment, arrow), (d) fetal biometry measurements (in centiles).

A 34‐year‐old primigravida with an unremarkable family history was referred at 23 weeks of gestation after multiple fetal anomalies were detected. Early fetal sonography and non‐invasive prenatal testing (NIPT) showed no abnormalities. However, significant fetal anomalies were detected by sonography at 23 weeks of gestation. The fetus presented with symmetric intrauterine growth restriction (IUGR, 3rd percentile) (Figure 1d), suspected corpus callosum hypoplasia (Figure 1a), fixed lower limbs with muscle atrophy (Figure 1b) and suspected retinal detachment (Figure 1c). Further sonographic examinations showed polyhydramnios, a small stomach, cardiomegaly, cerebellar hypoplasia and persistent hyperplastic primary vitreous (PHPV) in the left eye (Table 1A).

**TABLE 1 A:** Clinical data.

Case	Parental details			Gestation at diagnosis	Phenotypes (HPO terms)	Obstetric history	Family history	Outcome
1	Mother: Healthy	Age	34	23 weeks	Abnormality of prenatal development or birth (HP:0001197); intrauterine growth retardation (HP:0001511); limitation of joint mobility (HP:0001376); and abnormal vitreous humor morphology (HP:0004327) or cerebellar atrophy (HP:0001272).	Gravida 1 para 0	No primary findings for genetic disease; Non‐consanguineous	Pregnancy Termination at 24 weeks, no autopsy.
		Medical history	Healthy					
	Father: Healthy	Age	37					
		Medical history	Healthy					

## 2 Diagnostic Method

Initial routine diagnostic genetic testing, including numerical and structural chromosome analysis with GTG banding and trio‐based exome sequencing (WES), after amniocentesis in the 23rd week of gestation showed no pathogenic findings. Further testing and reanalysis of the WES data using a research‐based approach, identified compound heterozygous variants in the *ADSL* gene.

Functional confirmation included RNA sequencing (maternal blood) and adenylosuccinate lyase (ADSL) enzymatic activity assays were performed on parental peripheral blood mononuclear cells (PBMCs). Detailed protocols for all methods are provided in the Supporting Information S1.

## 3 Diagnostic Results and Interpretation

WES reanalysis identified NM_000026.4: c.1277G > A p.(Arg426His), inherited from the father, and NM_000026.4: c.597G > A p.(Lys199 = ), inherited from the mother. The paternal variant c.1277G > A is the most common variant reported in ADSL deficiency [1], based on the ACMG criteria classified as pathogenic (Table 1B). The maternal variant c.597G > A appears to be synonymous based on the genetic code, but the deep learning models SpliceAI and Pangolin predicted that it probably affects splicing (Δ score value of 0.8 with the SpliceAI model; this score ranges from 0 to 1 and is the largest change in splice prediction scores in the surrounding sequence context, with scores ≥ 0.8 interpreted as highly confident predictions) [2]. Whole blood RNA sequencing isolated from maternal blood confirmed aberrant splicing, resulting in a 56‐bp deletion ADSL (NM_000026.4): c.597_654delinsA p.(Gly200_Lys218del) and causing the degradation of the most transcripts via nonsense‐mediated decay (NMD) (Supporting Information S2: (A–B)). WES reanalysis did not identify any other pathogenic or likely pathogenic variants. A phenotype‐based gene selection was considered in the analysis of 22,000 genes. A total of 1926 genes associated with the HPO terms (Table 1A) were evaluated.

**TABLE 1 B:** Genetic findings.

Procedure (gest age)	Direct/culture?	Performed test	Secondary confirmatory test	Gene (name; REFSEQ)	Known disease (OMIM)	Variant	ACMG classify‐cation	Criteria applied	Inheritance & zygosity	Interpretation
Amnio‐centesis (23 w)	Direct	Trio‐WES	RNA‐seq; enzyme assay	ADSL (NM_000026.4)	ADSL deficiency (#103050)	Allele 1: c.1277G > A, p.(Arg426His)	Pathogenic	PS3: Functional evidence (reduced enzymatic activity) PM2_supporting: Absent/very rare in population databases PM3_very strong: Trans with other pathogenic variants PP3_moderate: Multiple in silico tools predict deleterious effect	Heterozygous; inherited from father	Most common pathogenic variant in ADSL deficiency; well‐established disease mechanism. ClinVar accession: VCV000002462.48
						Allele 2: c.597G > A, p.(Lys199 = )	Likely pathogenic	PS3: Aberrant splicing demonstrated by RNA‐seq, with transcript degradation via NMD PM2_supporting: Absent from gnomAD and other major population databases PM3: Compound heterozygous with known pathogenic variant PP3_moderate: SpliceAI and pangolin predict splicing impact (score ∼0.8)	Heterozygous; inherited from mother	Synonymous variant with functional impact; causes aberrant splicing with a 56‐bp deletion, leading to a frameshift and premature stop codon, followed by NMD. Submitted to ClinVar: SUB15554418

Enzymatic ADSL activity assays in the parental PBMCs showed reduced ADSL activity with both ADSL substrates: 13% of the control level for succinyladenosine monophosphate (SAMP) and 39% for succinylaminoimidazolecarboxamide ribotide (SAICAR) in the mother and 25% for SAMP and 35% for SAICAR in the father. These results support the pathogenicity of both variants, novel maternal c.597G > A and known paternal c.1277G > A (Table 1B).

## 4 Pregnancy Outcomes and Neonatal Findings

The parents were counseled by pediatric specialists because of the suspicion of arthrogryposis multiplex congenita. Due to the severity of the sonographic abnormalities, the parents opted to terminate the pregnancy at 24 weeks. The parents refused a postmortem examination.

## 5 Discussion

This case represents the first described and functionally validated severe prenatal presentation of ADSL deficiency (OMIM #103050) that resulted in termination of the pregnancy due to multiple severe sonographic abnormalities.

The detection of reduced ADSL activity in PBMCs of unaffected parents in our study, coupled with the knowledge that individuals with a pathogenic heterozygous variant in the *ADSL* gene do not accumulate SAICAr (unpublished results), supports the hypothesis that partial residual ADSL activity is sufficient to maintain purine homeostasis in postnatally diagnosed ADSL deficiency. In this condition, ADSL activity is reduced, but no purine deficiency has been documented [3], and the pathogenic mechanism is attributed to the accumulation of the toxic metabolite SAICAr [4].

In our case, one pathogenic variant significantly reduced ADSL activity and stability [5] and another caused NMD. Together, these variants likely resulted in a near‐complete loss of ADSL activity. ADSL is a key enzyme of *de novo* purine synthesis (DNPS), which is essential during early embryogenesis due to the high demand for nucleotides necessary for rapid cell proliferation, neurodevelopment, and tissue growth. Therefore, severe impairment of DNPS is expected to predominantly affect the central nervous system and fetal growth. The observed phenotype shows impaired cellular proliferation resulting in prenatal growth failure, neurodevelopmental degeneration, and secondary musculoskeletal and ocular abnormalities. This is more consistent with purine deficiency than metabolite‐mediated toxicity. This finding is crucial for providing appropriate genetic counseling, as such a phenotype may be incompatible with postnatal survival.

## Funding

This work was supported by the Ministry of Health of the Czech Republic (Grant NU23‐01‐00500) and by the project MULTIOMICS_CZ (Program Johannes Amos Comenius, Ministry of Education, Youth and Sports of the Czech Republic; Project ID: CZ.02.01.01/00/23_020/0008540), co‐funded by the European Union. Further support was provided by institutional programs of Charles University in Prague (UNCE 24/MED/022 and Cooperatio). RNA analyses were provided by the National Center for Medical Genomics (LM2023067), supported by the Ministry of Education, Youth and Sports of the Czech Republic. The funding bodies played no role in the study design, data analysis, data interpretation, or manuscript preparation.

## Ethics Statement

This study was approved by the Ethics Committee of the General University Hospital in Prague on 16 June 2022 (reference number: 39/22 Grant AZV VES 2023 1. LF UK).

## Consent

Written informed consent was obtained from all participants and/or their legal guardians for genetic testing and for publication of genetic and clinical data, including radiological images.

## Conflicts of Interest

The authors declare no conflicts of interest.

## Supporting information


Supporting Information S1

Supporting Information S2

## Data Availability

The data that support the findings of this study are available on request from the corresponding author. The data are not publicly available due to privacy or ethical restrictions.

## References

[1] G. Mastrogiorgio , M. Macchiaiolo , P. S. Buonuomo , et al., “Clinical and Molecular Characterization of Patients With Adenylosuccinate Lyase Deficiency,” Orphanet Journal of Rare Diseases 16, no. 1 (2021): 112, 10.1186/s13023-021-01731-6. 33648541 PMC7919308

[2] K. Jaganathan , S. Kyriazopoulou Panagiotopoulou , J. F. McRae , et al., “Predicting Splicing From Primary Sequence With Deep Learning,” Cell 176, no. 3 (2019): 535–548.e24, 10.1016/j.cell.2018.12.015. 30661751

[3] A. Jurecka , M. Zikanova , S. Kmoch , and A. Tylki‐Szymanska , “Adenylosuccinate Lyase Deficiency,” Journal of Inherited Metabolic Disease 38, no. 2 (2015): 231–242, 10.1007/s10545-014-9755-y. 25112391 PMC4341013

[4] O. Souckova , V. Skopova , V. Baresova , et al., “Metabolites of De Novo Purine Synthesis: Metabolic Regulators and Cytotoxic Compounds,” Metabolites 12 (2022): 1210, 10.3390/metabo12121210. 36557247 PMC9788633

[5] M. Zikanova , V. Skopova , A. Hnizda , J. Krijt , and S. Kmoch , “Biochemical and Structural Analysis of 14 Mutant Adsl Enzyme Complexes and Correlation to Phenotypic Heterogeneity of Adenylosuccinate Lyase Deficiency,” Human Mutation 31, no. 4 (2010): 445–455, 10.1002/humu.21212. 20127976

