# Supplementary material for: Severe Prenatal Presentation of Adenylosuccinate Lyase Deficiency Caused by a Synonymous ADSL Variant Inducing Aberrant Splicing
Source: Prenat Diagn. 2026 Jan 30;46(3):454–7. doi: 10.1002/pd.70087 (PMC12978515; doi:10.1002/pd.70087)
Supplement: Supplementary file 1 — Supporting Information S1 [file PD-46-454-s001.pdf]

Supplementary material S1

Detailed protocols for all methods

Chemicals

Succinylaminoimidazole carboxamide ribotide (SAICAR) was produced according to established methods (1). Succinyl adenosine monophosphate (SAMP), adenosine-5´-(a,b-methylene) diphosphate (MADP) and the remaining reagents, except where noted differently, were purchased from Sigma-Aldrich.

Exome sequencing

Whole-clinical exome sequencing was performed at the MGZ in Munich, Germany, using standard procedures. For targeted DNA enrichment of all coding and adjacent intronic regions, the Twist Human Comprehensive Exome + Mitochondrial Genome kit in combination with Illumina R sequencing technology was used. Bioinformatic analysis of the obtained sequence data for the detection of SNVs, small insertions/deletions (InDels <50bp), as well as copy number variations (CNVs) was performed using the software Varvis (Limbus Medical Technologies GmbH, Rostock, Germany). A mean coverage of 20–40x was achieved for >96% of the targeted coding regions, in accordance with the NGS guidelines of the German Society for Human Genetics (GfH, 2018). Sequence data with a mapping quality <20 were excluded from the analysis. The GRCh38 human reference genome was used for alignment. For phenotype-driven variant prioritization, Human Phenotype Ontology (HPO) terms, as stated in the clinical indication (2), were applied. Variant interpretation and classification followed the ACMG guidelines (3), considering parameters such as predicted functional impact, population allele frequency, inheritance pattern, and clinical correlation. After completion of the diagnostic analysis the sequencing data were transferred as BAM files to the collaborating

research group of the Institute of Human Genetics, University Hospital of Cologne, Germany. These files were further processed using an in-house developed next-generation sequencing (NGS) pipeline. An HPO-term phenotype-based re-analysis was conducted on a research basis. Following the identification of biallelic variants in the *ADSL* gene as a potential cause of disease, these variants were confirmed by Sanger sequencing as an independent validation method.

RNA isolation

Whole blood samples were collected in Tempus™ Blood RNA Tubes and total RNA was extracted using the Tempus™ Spin RNA Isolation Kit (Thermo Fisher Scientific Inc., Waltham, MA, USA), according to the manufacturer's instructions. RNA integrity was assessed using the RNA ScreenTape Assay on the Agilent 4200 TapeStation system (Agilent Technologies, Inc., Santa Clara, CA, USA), and all samples exhibited RNA Integrity Number (RIN) values greater than 8.0.

RNA sequencing

mRNA libraries were constructed using the KAPA mRNA HyperPrep Kit (F. Hoffmann-La Roche Ltd., Basel, Switzerland) and sequenced on the Illumina NovaSeq X platform (Illumina, Inc., San Diego, CA, USA). Sequence reads were processed and aligned using the Illumina DRAGEN RNA Pipeline (v4.1) - Illumina (2024). DRAGEN secondary analysis (Version 4.2, v4.1.23 Software) https://www.illumina.com/products/by-type/informatics-products/dragen-secondary-analysis.html, generating BAM files for downstream analysis. Alignment files were subsequently visualized using the Integrative Genomics Viewer (IGV, Broad Institute, Cambridge, MA, USA)(4).

Lysates Preparation

Isolation of peripheral blood mononuclear cells (PBMC) was performed from EDTA-blood using Histopaque-1077 according to manufacturer protocol. The pellets were dissolved in A buffer (10 mM Tris pH 8.2, 2 mM EDTA, 10 mM KCl, 1 mM DTT, and 4% glycerol) with Protease Inhibitor Cocktail Tablets (Roche Diagnostics GmbH, Mannheim, Germany) (for each 1×10^6 cells/ 50 µl of buffer was used), sonicated four times for 15s and centrifuged at 17 000g for 20 min at 4°C. Final protein concentration in cell lysates was measured by Bradford method.

ADSL Enzyme Catalytic Activity

ADSL catalytic activity was measured in PBMC of parents accordingly skin fibroblasts with slightly changes (5). Briefly, 1.5 mg/ml of PBMCs lysate was preincubated in 25°C for two hours, and then the reaction was performed at 37°C in A buffer with 0.31 mg/ml PBMCs lysate, 0.02 mg/ml MADP and SAMP (0.07 mg/ml) or SAICAR (0.1 mg/ml) substrate. The reaction was stopped after 40 min with SAMP, or 50 min with SAICAR substrate, resp. by 0,25 M perchloric acid and neutralized with 0,25 M $KHCO_3$. The final products adenosinemonophosphate (AMP) and aminoimidazolecarboxamide ribotide (AICAR) were analysed by HPLC using Shimadzu Nexera XR System with a PDA detector (1).

References

1. Zikanova M, Krijt J, Hartmannova H, Kmoch S. Preparation of 5-amino-4-imidazole-N-succinocarboxamide ribotide, 5-amino-4-imidazole-N-succinocarboxamide riboside and succinyladenosine, compounds usable in diagnosis and research of adenylosuccinate lyase deficiency. J Inherit Metab Dis. 2005;28(4):493-9.

2. Kohler S, Carmody L, Vasilevsky N, Jacobsen JOB, Danis D, Gourdine JP, et al. Expansion of the Human Phenotype Ontology (HPO) knowledge base and resources. Nucleic Acids Res. 2019;47(D1):D1018-D27.

3. Richards S, Aziz N, Bale S, Bick D, Das S, Gastier-Foster J, et al. Standards and guidelines for the interpretation of sequence variants: a joint consensus recommendation of the American College of Medical Genetics and Genomics and the Association for Molecular Pathology. Genet Med. 2015;17(5):405-24.

4. Thorvaldsdottir H, Robinson JT, Mesirov JP. Integrative Genomics Viewer (IGV): high-performance genomics data visualization and exploration. Brief Bioinform. 2013;14(2):178-92.

5. Kmoch S, Hartmannova H, Stiburkova B, Krijt J, Zikanova M, Sebesta I. Human adenylosuccinate lyase (ADSL), cloning and characterization of full-length cDNA and its isoform, gene structure and molecular basis for ADSL deficiency in six patients. Human Molecular Genetics. 2000;9(10):1501-13.
