# Supplementary material for: Severe Prenatal Presentation of Adenylosuccinate Lyase Deficiency Caused by a Synonymous ADSL Variant Inducing Aberrant Splicing
Source: Prenat Diagn. 2026 Jan 30;46(3):454–7. doi: 10.1002/pd.70087 (PMC12978515; doi:10.1002/pd.70087)
Supplement: Supplementary file 2 — Supporting Information S2 [file PD-46-454-s002.pdf]

Supplementary material S2: *RNA sequencing of whole blood reveals aberrant splicing due to the c.597G>A variant.*

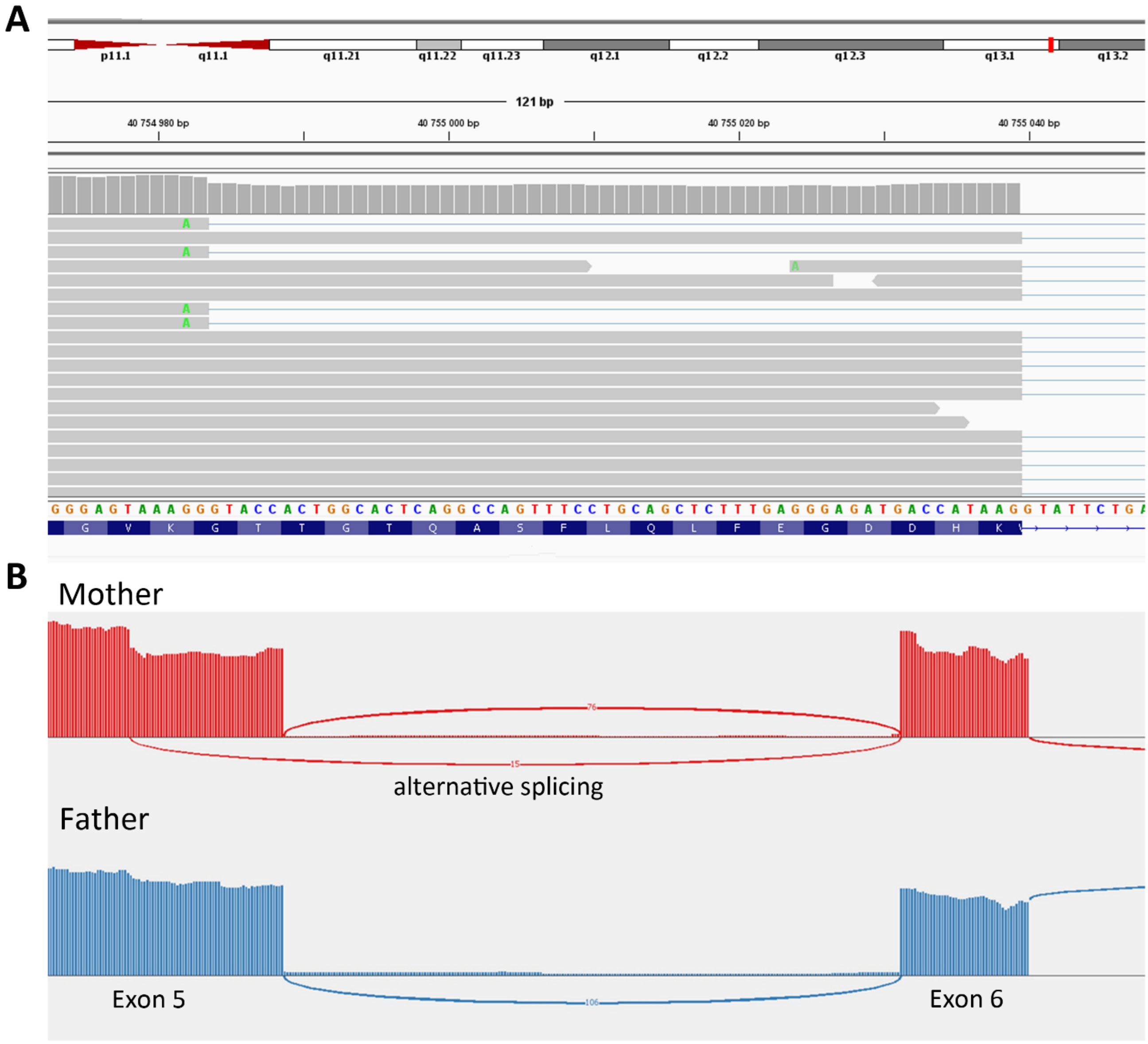

A. Visualization of a 56-bp loss in the transcript associated with the c.597G>A substitution. Aligned RNA-seq reads are shown in the Integrative Genomics Viewer (IGV), illustrating a 56-bp skipping in conjunction with a single-nucleotide substitution c.597G>A in *ADSL* gene. This splicing alteration is supported by split reads and reduced coverage over the affected region.

B. Visualization of alternative splicing in the *ADSL* transcript. Modified Sashimi plot displaying splice junctions between exon 5 and exon 6 in *ADSL* gene. In the mother's sample, two distinct splice events are detected. The alternative event occurs at approximately one-fifth the frequency of the canonical splicing and likely reflects partial exon skipping due to activation of a cryptic donor site introduced by the c.597G>A substitution. The relatively low abundance of the alternative transcript is consistent with nonsense-mediated decay (NMD). The father's sample shows only the canonical splicing pattern.
